# Outcomes of interventions to optimize linkage to HIV care and antiretroviral therapy (ART) initiation after HIV self-testing: A scoping review

**DOI:** 10.29392/001c.30064

**Published:** 2022-01-27

**Authors:** Patience A. Muwanguzi, Paul Kuodi Otiku, Blessings Gausi, Tom Denis Ngabirano, Scovia Nalugo Mbalinda, Mobolanle Balogun, Derrick Tembi Efie, Charles Peter Osingada

**Affiliations:** 1 School of Health Sciences, College of Health Sciences, Makerere University, Uganda; 2 Department of Community Health and Development, Faculty of Science, The Catholic University of Eastern Africa, Kenya; 3 School of Public Health and Family Medicine, Faculty of Health sciences, University of Cape Town, South Africa; 4 Department of Community Health and Primary Care, College of Medicine, University of Lagos, Nigeria; 5 Department of Public Health and Hygiene, Faculty of Health Sciences, University of Buea, Cameroon

**Keywords:** HIV self-testing, Linkage, Interventions, Outcomes

## Abstract

**Background:**

Human immunodeficiency virus (HIV) self-testing is an innovative solution to the problem of low HIV testing coverage. It can help in realizing the first “95” of the Joint United Nations Programme on HIV/AIDS targets in the HIV treatment cascade. However, there is limited information to guide how those who self-test and show positive results can successfully be linked to HIV care and treatment. Therefore, this scoping review aimed at synthesizing available evidence of the outcomes of intervention strategies to optimize linkage to HIV care after HIV self-testing.

**Methods:**

Our methodology followed Arksey and O’Malley’s methodological framework. Two independent reviewers screened and extracted data based on predetermined criteria. The databases searched included PubMed, EBSCOhost, Web of Science, Cochrane Library, Scopus, Mednar, and the International Clinical Trials Registry Platform.

**Results:**

A total of 4809 records were retrieved. After full-text screening, 14 studies met the inclusion criteria for the review. The intervention strategies reported were classified into four main categories: technology-assisted interventions, innovative HIV self-testing kits distribution mechanisms, financial incentive, social entrepreneurship models, and the use of key community opinion leaders and social media influencers. This scoping review found men who have sex with men as the main recipients of the interventions to improve the rate of linkage to HIV care following HIV self-testing. Studies that met the inclusion criteria reported mixed findings on the outcomes of interventions to improve the rate of linkage to HIV care following HIV self-testing. Financial incentives, interventions leveraging technology, and key opinion leaders were the most effective strategies.

**Conclusions:**

Given that the included studies did not employ a uniform system of measurement of effectiveness, there is a need for identification of standardized definitions and clear indicators for evaluating linkage to care and antiretroviral therapy (ART) initiation following HIV self-testing.

Human immunodeficiency virus (HIV) self-testing is a process in which a person collects his or her own specimen (oral fluid or blood), using a simple rapid test and then performs an HIV test and interprets the result, often in a private setting, either alone or with someone he or she trusts.^1^ HIV self-testing is an innovative solution to the problem of low HIV testing coverage and can be performed and distributed in several contexts within and outside health facility environments. HIV self-testing can help in realizing the first “95” of the Joint United Nations Programme on HIV/AIDS 95-95-95 goals for 2030 targets in the HIV treatment cascade. This is by enabling individuals who have never tested and are not currently reached by the existing HIV testing and counselling services to conveniently obtain information on their status.^2^ Evidence from studies have shown promising results from HIV self-testing interventions with reports of high acceptability (74–96%) among men who have sex with men, young people, health workers, and couples, and has a high potential to increase HIV testing coverage.^3–8^

Despite the existing evidence of high uptake and acceptability, linkage to care among those who receive reactive HIV self-test results remains a challenge.^9–12^ Additionally, there is limited evidence available to guide how those who self-test and show positive results could be linked to HIV care and treatment. Interventions, some leveraging on technology such as mobile applications, short messaging service reminders, and others employing innovative ways of healthcare delivery, have been applied to help link those who test positive during HIV self-testing to routine HIV care.^13,14^ Findings from these interventions, including those from pilot programs aiming to increase linkage to care and HIV treatment, have shown promising results in various settings and among different population segments. The extent of effectiveness of these interventions requires further evaluation through the collation and synthesis of existing evidence to inform the development of normative HIV self-testing and treatment guidelines. Therefore, this scoping review aimed at synthesizing available evidence of intervention strategies to facilitate linkage to HIV care after HIV self-testing.

## METHODS

### STUDY DESIGN

The methodology of this scoping review follows the framework described by Arksey and O’Malley^15^ and modified by Levac et al,.^16^ The hybrid framework involves six steps for undertaking a scoping review, as described below.

#### STEP 1: IDENTIFYING THE RESEARCH QUESTION

Evidence suggests that linkage to HIV care after HIV self-testing remains a challenge.^17,18^ Several interventions have been designed to optimize sequential HIV treatment after HIV self-testing, however, there is no documented aggregated evidence of the outcomes of these strategies. Therefore, this scoping review was guided by the research question “What are the outcomes of the intervention strategies designed to improve linkage to HIV care and treatment after HIV self-testing?”.

Specifically, the review seeks to answer the following questions:

1. Who is the target population for interventions aimed at improving the rate of linkage to care and antiretroviral therapy initiation after HIV self-testing?
2. What type of interventions are being implemented to improve the rate of linkage to care and antiretroviral therapy initiation after HIV self-testing?
3. What are the outcomes of the interventions aimed at improving the rate of linkage to HIV care and antiretroviral therapy initiation after HIV self-testing?
4. What interventions are effective in improving the rate of linkage to care and antiretroviral therapy initiation after HIV self-testing?

#### STEP 2: SETTING INCLUSION AND EXCLUSION CRITERIA

We applied the PCC (Population, Concept, and Contexts) framework to define the inclusion criteria as proposed by Peters et al,.^19^ The PCC framework is appropriate for scoping reviews because it allows for the inclusion of studies that do not contain information about outcomes or comparator groups.

Accordingly, eligible populations for this scoping review included all categories of persons who could benefit from HIV self-testing, including men who have sex with men, health workers, pregnant women receiving antenatal care, partners/couples, female, and male sex workers, among others. Important concepts for the scoping review included the type of intervention implemented, the approach to HIV self-testing used, approaches to linkage to care and treatment after HIV self-testing, and how the rate of linkage to care after HIV self-testing was determined.

Studies for inclusion in the review fulfilled the following criteria: (1) Reported specific approach(es) to HIV self-testing; (2) Reported on specific intervention(s) to improve the rate of linkage to care and treatment after HIV self-testing; (3) Reported quantifiable outcome(s) of the intervention to improve the rate of linkage to care after HIV self-testing.

For the study design criteria, the scoping review included the following types of studies: randomised controlled trials, pragmatic trials, non-randomised controlled trials, and observational studies, with or without controls. Non-primary studies, such as different types of reviews, conference proceedings, book chapters, guidelines, and any other form of aggregated evidence, were excluded from the review despite being considered.

#### STEP 3: SEARCHING AND SELECTING THE EVIDENCE

The literature search was guided by the research questions and the Population, Concept, and Context’s framework criteria described above. A search strategy was developed and applied to different databases in accordance with the Peer Review of Electronic Search Strategies guidelines.^20^ The search strategy was piloted on the PubMed database. Search terms and free-text words were combined using the Boolean operators ‘AND’ and ‘OR’, such as (linkage OR retention, HIV OR AIDS) AND (self-testing OR “home test” OR “unsupervised test”) AND (interventions OR trials OR pragmatic trials OR implementation science). Search terms also included other controlled descriptors such as Medical Subject Headings and their synonyms. The search strategy was applied to PubMed and modified for use in other databases. Table S1 in the online supplementary document.

A comprehensive literature search was carried out on the following electronic databases: MEDLINE (via PubMed), Cochrane Library including the Cochrane Central Register of Controlled Trials, Scopus, Cumulative Index to Nursing and Allied Health Literature (CINAHL) and the International Clinical Trials Registry Platform. Additionally, relevant grey literature was sourced from Mednar for potentially eligible articles. No language and geographical restrictions were applied during the search.

After obtaining full records, potentially eligible articles were screened at two levels: title and abstract screening and full-text screening each time, selecting eligible studies based on predefined inclusion/exclusion criteria. Two reviewers independently screened the titles and abstracts of all retrieved records from the search output. Articles meeting the inclusion criteria were further subjected to a full-text assessment for eligibility. Disagreements on article eligibility were resolved through consensus between the first and the second author. Further disagreements were resolved by seeking the opinion of two other reviewers.

#### STEP 4: CHARTING THE EVIDENCE

The first and second reviewers independently abstracted and recorded the data from eligible articles using a data abstraction tool. The abstraction tool includes four domains: (1) study details (article title, authors; country(setting); publication year); (2) design and methods (Design; study objective or research question; sample characteristics (e.g., sample size, sex, age, ethnicity, population groups, follow-up duration; validation of measures; statistical analyses); (3) study outcomes (HIV self-testing approach, linkage to care approach, type of intervention) and (4) study conclusions.

The second author combined the abstracted data and carried out the analysis. The first author and third author double-checked the entered data for completeness and verified the accuracy of the analysis. The other reviewers checked the data for accuracy and consistency with the study protocol. For validity, fourth author re-ran the search strategy and followed the same process to confirm the findings.

#### STEP 5: SYNTHESIZING AND REPORTING THE EVIDENCE

The reporting of the findings of this review follows the Preferred Reporting Items for Systematic reviews and Meta-Analyses extension for Scoping Reviews guidelines.^21^ Table S2 in the online supplementary document. The data were charted and summarised narratively, and evidence synthesised based on themes that emerged from the charted data. Quantitative evidence was aggregated using appropriate summary statistics and methods. The overall assessment of the synthesised evidence is presented narratively rather than quantitatively.

#### STEP 6: CONSULTATION WITH STAKEHOLDERS

We engaged experts in HIV programming to obtain relevant grey literature not captured by the literature search. Other stakeholders provided guidance with the identification of important concepts and the interpretation of the review findings.

### ETHICS AND DISSEMINATION

Given that this scoping review utilised publicly available literature, the requirement for ethical approval was waived. This review provides synthesised evidence that may be usable by health policymakers and HIV programmers

## RESULTS

### SELECTION OF STUDIES

**Figure 1.**
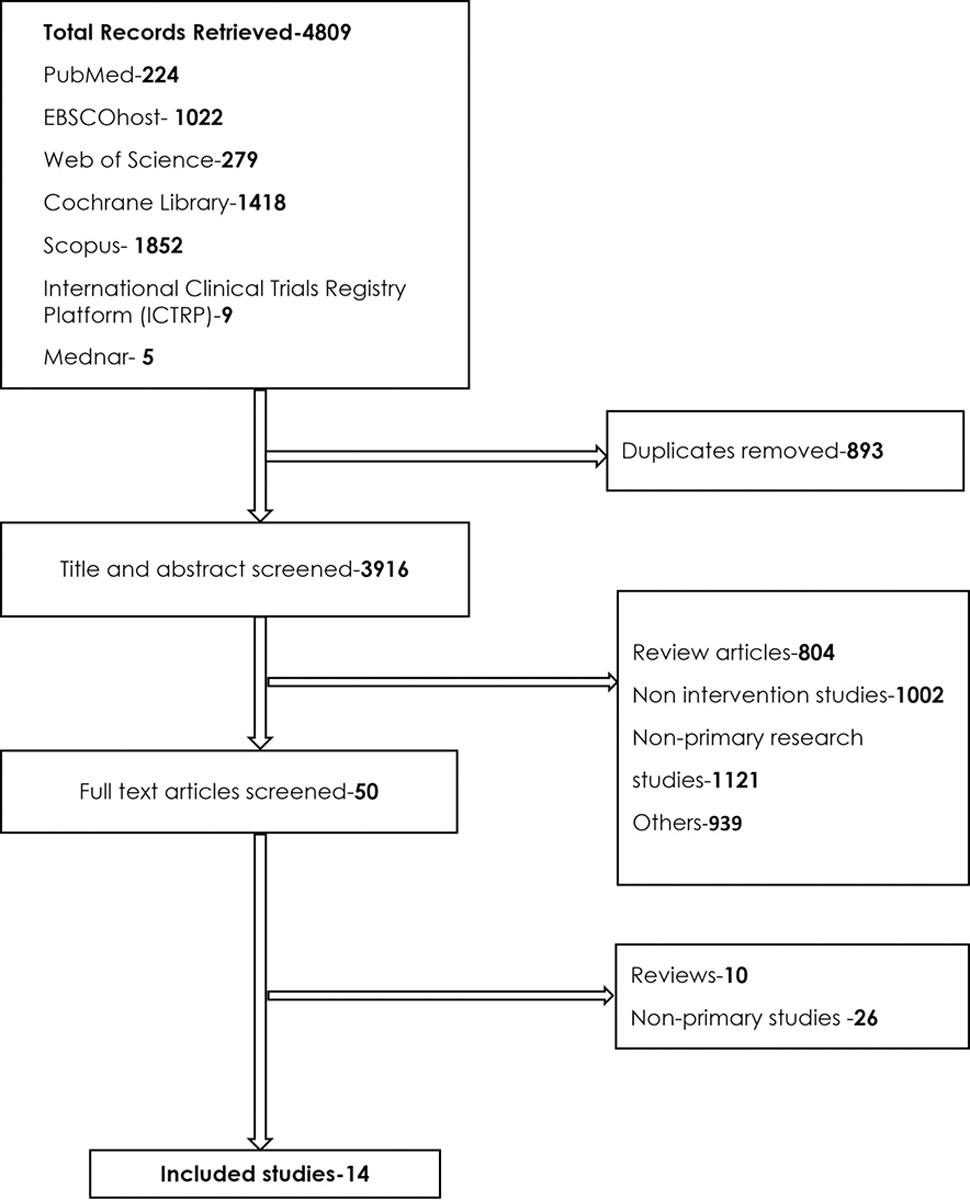
Flow diagram showing selection of eligible articles

A total of 4809 records were retrieved. Two hundred and twenty-four records were retrieved from PubMed database, 1022 from EBSCOhost, 279 from Web of Science, 1418 from Cochrane Library, 1852 from Scopus, 9 from the International Clinical Trials Registry Platform and 5 from Mednar grey literature database. After the removal of 893 duplicates from the retrieved records, 3916 studies were subjected to title and abstract screening. After completion of title and abstract screening, 3866 records were excluded for various reasons as indicated in Figure 1.

Eight hundred and four studies were excluded because they were review articles, 1002 were excluded because they were non-intervention studies, 1121 were excluded because they were **non**-primary research studies, while 939 studies were excluded for other reasons (no clear quantifiable outcomes, did not report the outcome of interest for the review, among other reasons shown in Figure 1.

After the completion of title and abstract screening, 50 articles met the inclusion criteria and were further subjected to full-text screening. Ten studies were found to be reviews during the full-text screening, while 26 studies were non-primary studies and were, as a result, excluded. After the full-text screening, 14 studies met the inclusion criteria for the review. Figure 1 shows a summary of steps in the study selection process, from database to screening and final selection of included studies.

### CHARACTERISTICS OF INCLUDED STUDIES

Eight^22–29^ of the included studies were randomised controlled trials, three^13,14,30^ were cross-sectional studies, two^31,32^ were cohort studies and one^33^ was a discrete choice experiment. Seven studies were carried out among men who have sex with men^14,25,28,31,32^, while the rest of the studies were conducted among other adult men, and among women receiving antenatal care.^22–24,26,27,29,33^ Table 1 shows a summary of the characteristics of the included studies.

**Figure 2.**
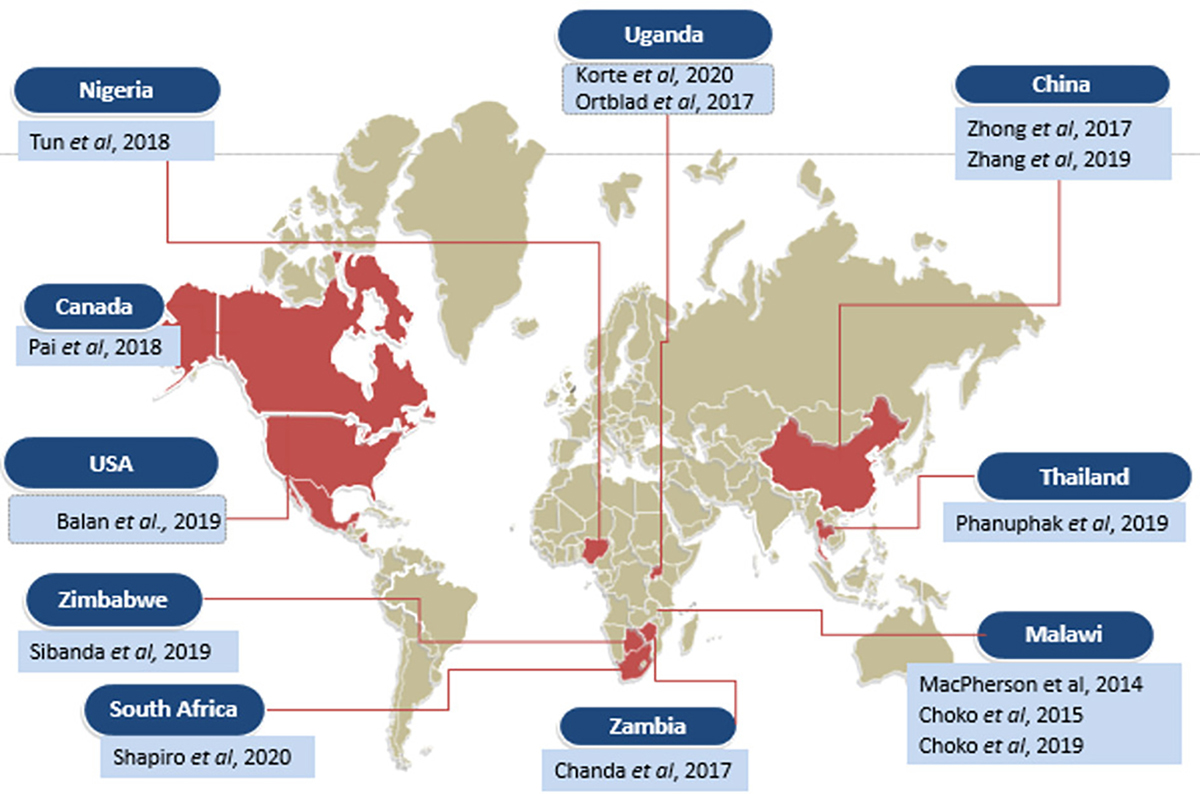
Geographical distribution of included studies

In terms of setting, nine studies were conducted in Africa^22–27,29,31,33^, two in the United States and Canada^13,14^ and 3 in Asia.^28,30,32^ The geographical distribution of the included studies is shown in Figure 2.

### INTERVENTION STRATEGIES TO IMPROVE LINKAGE TO CARE AND TREATMENT

Three included studies reported interventions leveraging on technology to improve the rate of linkage to care following HIV self-testing. Mobile phone applications and supervised online testing were the two technology-assisted interventions that were reported^13,14,32^ to support linkage to HIV care following self-testing. Clients voluntarily followed prompts outlined in the mobile application or online platforms to guide the next steps in accessing care following a positive HIV self-test.

Seven studies^22–26,29,33^ reported different innovative HIV self-testing kit distribution approaches as the intervention implemented to improve HIV self-testing. Door-to-door, community outreaches or antenatal care and other targeted health facility sites were the distribution mechanisms used. Following a reactive self-test, lay counsellors, peer educators, and health workers in the community or at health facilities helped with linkage to HIV care. In some instances, clients were offered self-referral cards with directions for seeking care following a positive HIV-self test.

Two included studies^27,30^ reported the use of an entrepreneurship model in the delivery of HIV self-testing kits. The models used financial incentives to encourage uptake of HIV self-testing and linkage to care following a positive HIV test.

The last category of intervention strategies relied on key community opinion leaders and social media influencers.^28,31^ These champions used their vantage positions to inspire HIV self-testing and linkage to HIV care following positive HIV self-tests. Table 2 below summarises the interventions implemented and their outcomes.

### OUTCOMES OF INTERVENTION STRATEGIES TO IMPROVE LINKAGE TO CARE AND TREATMENT

The targeted strategies had mixed outcomes. Smart mobile applications were100% successful in linkage to care for participants who tested positive for HIV.^13,14^ The online health worker supervised HIV self-testing and linkage to care platform was successful in linking slightly more than half of the participants who tested positive for HIV.^32^ SMS and phone call reminders were largely successful in increasing the rates of linkage to care and ART initiation following reactive self-tests.^33^

Innovative HIV self-test kit-distribution mechanisms registered some success with the linkage of individuals who tested positive for HIV during HIV self-testing HIV self-test. The community distribution of HIV self-test kits by lay health workers and volunteer counsellors was successful in achieving more than 50% linkage to care.^22,23,25^ Home distribution of HIV self-test kits was found to lead to higher percentages of linkage to HIV care and was found to be superior to facility-based linkage to HIV care.^29^ Likewise, the provision of HIV self-test kits for the partners of women attending antenatal care was associated with a higher successful linkage rate than routine HIV counselling and testing approaches.^24^ In contrast, the direct provision of oral HIV self-test kits and a coupon to facilitate health facility collection of HIVT kits and linkage to HIV care was found to have a less impact on the rate of linkage to HIV care than the routine HIV counselling and testing.^26^

Two studies reported the application of financial and social entrepreneurship models to improve the rate of linkage to HIV care following HIV self-testing. This approach achieved a 100% success rate in both studies.^27,30^ Key opinion leaders and social media influencers achieved successful linkage to care and ART initiation for more than 90% of the clients with reactive HIV self-tests.^28,31^

## DISCUSSION

Available evidence from the review reported four different categories of interventions aimed at improving HIV self-testing and linkage to HIV care after reactive self-tests. The first category of interventions leveraged digital and mobile phone technology to improve the rate of linkage to HIV care and uptake of HIV self-testing. Mobile phone applications, social media, short message services and direct phone calls were the common strategies employed. The second category of interventions applied innovative methods of HIV self-testing kits distribution in the community and at the facility and utilised existing lay health workers, volunteer counsellors, and community health counsellors to link participants who tested positive for HIV for confirmatory testing and ART initiation. The other broad category of interventions used financial incentives and social entrepreneurship models. Finally, several strategies involved partnerships with key community and social media influencers to encourage the uptake of HIV self-testing and to improve the rate of linkage to HIV care following reactive self-tests.

Interventions to improve the rates of HIV self-testing and linkage to care and ART initiation after self-testing mostly targeted men who have sex with men, transgender persons, commercial sex workers, and women receiving antenatal care and their partners. Other segments of the population reported by included studies include populations adult population in the communities and the health facilities.

### LIMITATIONS

This scoping review has been limited by the population reported by the included studies. men who have sex with men and transgender women that has been widely reported by included studies do not represent the general population as they are a population with unique characteristics.

## CONCLUSIONS

This scoping review identified men who have sex with men as the main population currently targeted with interventions to improve the rates of linkage to HIV care and ART initiation following HIV self-testing. Most of the studies that met the inclusion criteria were from the African continent, highlighting the research interest in this topic and its relevance in the region. Additionally, the studies that met the inclusion criteria reported mixed findings on the outcomes of interventions to improve the rate of linkage to HIV care following HIV self-testing. Financial incentives, interventions leveraging on technology, and key opinion leaders were the most effective interventions reported. Interventions such as providing the coupons to facilitate HIV service access had the least impact on the rates of linkage to care and ART initiation following reactive HIV self-tests. Concrete conclusions on the impact of interventions to improve the rate of linkage to HIV care following HIV self-testing are limited by the target population that is reported by most of the included studies in this scoping review. This highlights the need for further studies regarding linkage to HIV care among broader populations undergoing HIV self-testing interventions. There is also a need for a standardised definition and clear indicators for determination of the rates of linkage to care and ART initiation, as the included studies did not employ a uniform system of measurement.

## Supplementary Material

Table S1

Table S2

## Figures and Tables

**Table 1.** Characteristics of included studies

First Author, Year	Design	Setting/Country	Population	Intervention	Outcomes	Follow up Duration
Balán et al, 2019^14^	Cross-sectional pilot study	New York, USA	Men, transgender women-men who have sex with men	SMARTtest: A Smartphone App to Facilitate HIV and Syphilis Self and Partner-Testing, Interpretation of Results, and Linkage to Care	App assisted use of the HIVST kits provided self-reported test results	NA
Tun et al, 2018 ^31^	Cohort study	Lagos, Nigeria	men who have sex with men, (17–59) years	Using key opinion leaders to reach men who have sex with men with HIVST kits	Use of the HIVST kits provided and their experience	3 months
Sibanda et al, 2019^33^	Discrete Choice Experiment	2 rural districts, Zimbabwe	Men and women ≥16 years old (mean age 37.10)	Door-to-door HIVST kits distribution by community volunteers	Method of HIVST kits distribution preferences.	NA
Phanuphak et al, 2019^32^	Cohort study	Bangkok Metropolitan Thailand	men who have sex with men and transgender workers	Online, supervised, HIV self-testing.	HIV testing preference and experience, linkage to care	12 months
Shapiro et al, 2020^22^	RCT	Kwa Zulu Natal, South Africa	Adult men Median Age (28)	Lay counsellors distributed HIVST kits, conducted a live demonstration of how to use and interpret both test kits, and provided cell-phone videos demonstrating kit use,	Reported HIVST, linkage to care	3 months
MacPherson et al, 2014^29^	RCT	Blantyre, Malawi	Adult male and female (16 years and above)	Optional Home Initiation of HIV Care Following HIV Self-testing	Cumulative incidence of ART initiation, accepting HIV self-testing kit, reporting a positive HIV self-test result to counsellors	6 months
Korte et al, 2020^24^	RCT	Peri urban Hospitals Uganda	Male partners of women attending ANC. Age (yr), mean (25.2)	provided women with education and up to 4 HIVST kits to deliver to their male partners and other adults in the household.	Woman’s self-report of male partner HIV testing, man’s self-report, and measure combining self-report from both partners	3 months
Zhong et al, 2017^30^	Cross sectional survey	Guangzhou, China	males 18 years men who have sex with men	Social-Entrepreneurship Model to Promote HIV Self-testing and linkage to care among men who have sex with men	Prevalence of HIVS kits use, linkage to care frequency	1 month
Pai et al, 2018^13^	Cross-sectional study	Montreal, Canada	18 years or older, self-identified as men who have sex with men	Unsupervised Smart App-Optimized HIV Self-Testing Program	self-test conduct; self-test interpretation; and linkages to care	NA
Choko et al, 2015^23^	RCT	Blantyre, Malawi	Adults (>16 y old) residents	comparing health outcomes between 14 clusters randomised to HIVST and 14 clusters randomised to routine (facility-based) HTC	Uptake of testing, accuracy, linkage into care,	2 years
Chanda et al, 2017^25^	RCT	Kapiri Mposhi, Chirundu, and Livingstone, Zambia	18 years of age or older men who have sex with men	Peer educators distributed 2 HIV self-test kits plus instructions on its use	Actual use of the HIV self-tests, self-report their HIV status at the 4-month visit.	4 months
Ortblad et al, 2017^26^	RCT	Kampala, Uganda,	18 years or older, FSWs	Direct provision, facility collection of HIV self-tests versus standard of care	intimate partner violence (IPV), HIV self-test use and linkage to care	4 months
Choko et al, 2019^27^	RCT	Blantyre, Malawi	women attending antenatal care	HIV self-testing alone or with additional interventions, including financial incentives and linkage to care	Proportion of male partners who tested for HIV and linked into care	1 month
Zhang et al, 2019^28^	Pragmatic trial	China	Men who have sex with men	HIV Self-Testing Programs to men who have sex with men delivered by Social Media Key Opinion Leaders and Community-Based Organizations	Number of HIVST respondents recruited by each investigator in SMKOL-strategy and CBO strategy	1 year

MSM- Men having Sex with Men, RCT- Randomised Controlled Trial, ANC- Antenatal Care, TGW- Transgender Women, FSWs- Female Sex Workers, CBO- Community Based Organisation, SMKOL- Social Media Key Opinion Leaders, HTC- HIV Testing and Counselling

**Table 2.** Outcomes of interventions to improve the rate of linkage to care following HIV self-testing

Mode	Interventions	Outcomes
**Technology assisted interventions**	Balan et al., 2019- linkage was achieved through a SMARTtest mobile app that allowed users to filter nearest clinic locations by their zip codes, their operating hours, website links and contact information.	SMARTtest app achieved 100% linkage for all 60 participants and their partners
	Pai et al., 2018- HIVSmart mobile app where participants were linked to further care by dedicated counsellors after HIVST.	All participants 451 (100%) were successfully linked to HIV counselling and other HIV care services
	Phanuphak *et al.,* 2020- compared routine HCT with health worker supervised online HIVST, and onsite health worker supervised HIVST.	Only 18 out of 60 (52.8%) HIV positive participants were successfully linked to care in the online and onsite supervised HIVST and linkage to care approaches. The routine HCT approach proved superior achieving 84% linkage to care and ART initiation.
	Sibanda et al., 2019- Discrete choice experiment where different scenarios of linkage to care following HIVST after test kit distribution were assessed. SMS linkage support, phone call linkage support, in person linkage support and extended clinic operating hours were some of the scenarios assessed for their impact on linkage to care	SMS support increased linkage to HIV care by 4.9 percentage points, phone call increased linkage by 6.5%, in person linkage support increased linkage by 6.7% and extending clinic operating hours increased linkage by 2.5%.
**Innovative HIV self-testing kits distribution mechanisms**	Shapiro et al., 2020- For linkage, counsellors provided post-test counselling and referral and SMS reminders for those who delayed sending results.	Among 274 men with positive HIVST results, 187 (68%) were successfully linked to ART and other HIV care services.
	Chanda *et al.,* 2017- Compared the routine HCT with peer educators’ distribution of oral HIVST kits to participants, and the collection of HIVST kits from a clinic or pharmacy using a coupon.	Among participants reporting an HIV positive result after the HIVST, linkage to care was non significantly lower among HIVST groups compared to the routine HCT. Routine HCT- 72/84 (85.7%), peer educator HIVST kits delivery- 53/74 (71.6%) and coupon clinic/pharmacy collection of HIVST kits- 59/77 (76.6%).
	Ortblad et. al.,2017 - Compared the routine HCT with direct provision of oral HIVST kits to participants, and the collection of HIVST kits from a health facility using a coupon.	Among participants who carried out HIVST, linkage to care was non significantly lower among HIVST groups compared to the routine HCT. Routine HCT- 37/294 (12.6%), direct provision of HIVST kits - 27/260 (10.4%) and coupon health facility collection of HIVST kits- 37/289(12.8%).
	Choko et al., 2015- Trained volunteer counsellors oral HIVST kits to adults in the community. Linkage was achieved through a self-referral card given to each participant. The card allowed direct access to the study clinic. Volunteer counsellors provided post-test counselling and referral.	Successful linkage was achieved with 524 participants out of 930 participants (56.3%).
	MacPherson et al., 2014- Routine facility-based care was compared with optional home initiation of HIV care (including 2 weeks of ART if eligible) after HIVST.	A significantly greater proportion of adults in the home base care group were linked to care and initiated ART, 2.2% (181/8194) compared with the routine facility-based group, 0.7% (63/8466).
	Korte et al., 2020- compared the routine care and the intervention where women attending antenatal care were each provided with up to 4 OraQuick HIVST kits. Linkage to care was achieved through providing the women with referral resource containing list of clinics and HIV testing sites, contact information of nurse counsellors and follow-up dates at 1-month and at 3 months	26 HIV positive men were identified in the intervention arm among whom 23% (6/26) were successfully linked to HIV care compared to 67% (4/6) from the routine care.
**Financial incentives and social entrepreneurship models**	Zhong et al., 2017- implemented a social entrepreneurship model. Participants bought the HIVST kits at $30 USD which was refunded upon successfully performing the HIVST and relaying the results to health workers. Linkage was achieved through a phone call to all participants with positive HIVST.	8 cases turned positive after HIVST. All cases were successfully linked to further HIV care.
	Choko et al., 2019- compared five scenarios of HIVST distribution some hinged on financial incentives: Briefly, the interventions implemented include: women attending antenatal care were provided with 2 HIVST kits for their partner, women attending antenatal care were provided with 2 HIVST kits for their partner plus incentive of $3 USD, women attending ANC were provided with 2 HIVST kits for their partner plus $10, women attending antenatal care were provided with 2 HIVST kits for their partner plus a 10% chance of winning $30 USD in a lottery, women attending antenatal care were provided with 2 HIVST kits for their partner and phone call reminder to the women’s partners.	Linkage to care following HIVST increased substantially using financial incentives. Linkage to care within 28 days was 13% with routine care, 40.9% with delivery of test kits only, 51.7% with HIVST kits and financial incentives, 18.6% with HIVST kits plus lottery, and 22.3% with HIVST plus phone reminder.
**Use of key community opinion leaders and social media influencers**	Tun et al., 2018 - Distributed oral HIVST kits to MSM through key opinion leaders.	Fourteen MSM tested positive for HIV after the HIVST. All 14 sought presented themselves at the health facility for test results confirmation and were successfully initiated on ART.
	Zhang et al., 2020- compared linkage to care, antiretroviral treatment, and cost of HIVST among MSM recruited by social media key opinion leaders and by community-based organisations. Linkage was achieved by peer navigators who were trained volunteers from the local community-based organisation that paired up with the participants to ensure successful linkage	The proportion of MSM in the CBO group initiating ART after linkage to care was lower than that in the social media key opinion leaders’ group (94.4% (31/33) compared to 29.0% (29/100)).
