# Supplementary material for: Outcomes of interventions to optimize linkage to HIV care and antiretroviral therapy (ART) initiation after HIV self-testing: A scoping review
Source: J Glob Health Rep. Author manuscript; Available in PMC 2023 Jun 14. (PMC10263187; doi:10.29392/001c.30064)
Supplement: Table S1 [file NIHMS1852272-supplement-Table_S1.pdf]

Table S1: Sample search strategy

Search strategy for use in PUBMED		
**Query**	**Fields**	**Search term**
#1	All	linkage OR retention, HIV OR AIDS
#2	All	self-testing OR “home test” OR “unsupervised test”
#3	All	interventions OR trials OR pragmatic trials OR implementation science
#4	#1 AND #2 AND #3	
